# Prospective medium-term results of multimodal pain management in patients with lumbar radiculopathy

**DOI:** 10.1038/srep28187

**Published:** 2016-06-16

**Authors:** A. Benditz, M. Madl, M. Loher, J. Grifka, D. Boluki, O. Linhardt

**Affiliations:** 1 Orthopädische Klinik für die Universität Regensburg, Asklepios Klinikum Bad Abbach, Kaiser-Karl-V-Allee 3, 93077 Bad Abbach, Germany; 2 Orthopädisches Versorgungszentrum München Innenstadt, Sendlinger Str. 14, 80331 München, Germany; 3 Caritas Krankenhaus St. Josef, Landshuter Strasse 65, 93053 Regensburg, Germany; 4 OZA München, Englschalkinger Str. 12, 81925 München, Germany

## Abstract

Lumbar radiculopathy is one of the most common diseases of modern civilisation. Multimodal pain management (MPM) represents a central approach to avoiding surgery. Only few medium-term results have been published in the literature so far. This study compared subjective and objective as well as anamnestic and clinical parameters of 60 patients who had undergone inpatient MPM because of lumbar radiculopathy before and 1 year ±2 weeks after treatment. The majority of patients were very satisfied (35%) or satisfied (52%) with the treatment outcome. Merely 8 patients commented neutrally and none negatively. The finger-floor distance had decreased significantly (p < 0.01), and 30 patients (50%) had shown improved mobility of the spine after therapy. The need for painkillers had also been significantly reduced after 1 year. The arithmetical average of pain on a visual analogue scale was 7.21 before treatment, which had significantly decreased to 3.58 at follow-up (p < 0.01). MPM is an effective approach for treating lumbar radiculopathy by mechanical nerve root irritation. Therefore, in the absence of an absolute indication for surgery or an absolute contradiction for MPM, patients should first be treated with this minimally invasive therapy.

In modern societies, lumbar radiculopathy and sciatica have become an increasing problem. Although all age groups are affected, elderly people are the largest population presenting with lumbar spine diseases. Lumbar radiculopathy may be caused by a herniated disc, disk protrusion or sciatica. Irritation or compression of the sciatic nerve often results in radicular pain along a specific dermatome and pseudoradicular pain. Such pain is often associated with claudicatio spinalis which decreases walking distances.

Finding the right treatment is important to these patients, and surgery should only be considered as a last resort. On the other hand, long-term pain must also be avoided to prevent pain chronification. Most of the many conservative treatment options available are not standardised and thus difficult to compare. For lumbar radicular pain syndrome, injections are an important treatment modality for reducing the irritation of affected anatomical structures^1^,^2^. Injection therapy is often accompanied by multimodal treatment such as physical therapy and psychological counselling. Over the past years, multimodal pain management (MPM) consisting of inpatient injection therapy has been improved by the results obtained in new trials and by the growing experience of physicians^3^. MPM has been shown to be a very beneficial treatment option for avoiding surgery in patients with radicular nerve route compression^4^,^5^,^6^. Therefore, all conservative treatment options should be explored in the absence of an absolute indication for surgery^1^,^7^.

## Aim of the study

The study aimed at proving the positive mid-term effect of a multimodal inpatient therapeutic concept based on drug injections for patients with lumbar radiculopathy.

## Methods

This clinical study with a non-randomised unblinded prospective design included male and female patients with lumbar radiculopathy aged 29 to 79 years who had been treated according to a multimodal therapeutic concept at the Department of Orthopaedics of the University Medical Centre Regensburg between April 2009 and May 2010. Participation in the study was voluntary. Inclusion criterion was radicular pain arising from one specific nerve root diagnosed by the presence of either the Lasègue sign, pain radiating distal to the knee or clearly attributable motoric failure. In addition, patients had to have participated in at least five psychological sessions during therapy. A clear indication for MPM had to exist, and an absolute indication for surgery had to be excluded.

Exclusion criteria were post-discectomy syndrome, rheumatic or inflammatory spinal disorders, tumours with spinal involvement and congenital spinal deformities. For ethical reasons, no control group was formed because such patients would have received placebo injections instead of the effective drugs. The study was approved by the Ethics Commission of the University of Regensburg (21.04.2010, Aktenzeichen 10-101-0061) and carried out in accordance to approved guidelines of the Helsinki Declaration of 1975. A written informed consent was obtained from all subjects. The study is registered in the DRKS with number DRKS00010257 (WHO register).

### Patients

**Figure 1:**
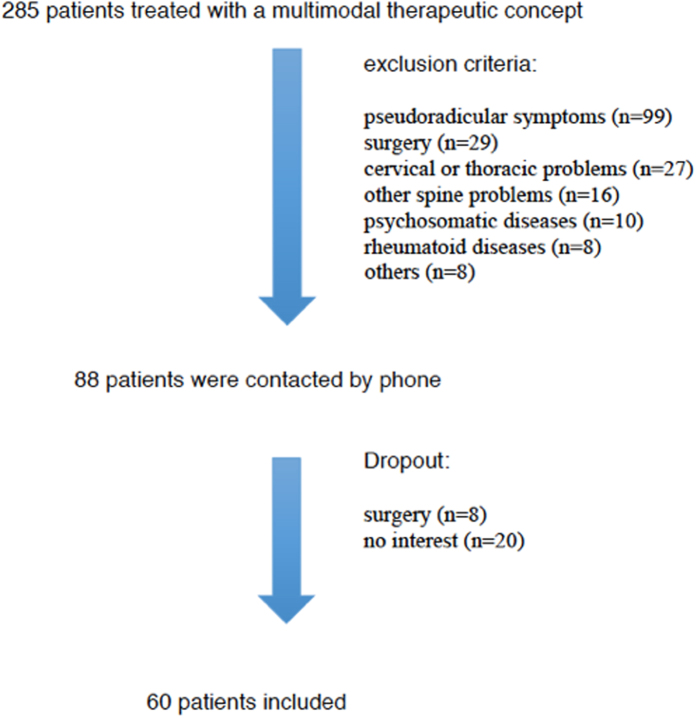
Flowchart of patient inclusion.

88 of initially 285 patients remained after evaluation of the patient files for exclusion criteria. After a telephone interview, further 28 patients were excluded, so that 60 volunteers remained in the study (Fig. 1, Table 1).

### Intervention

The duration of the treatment was 8–12 days (mean 10.8 days) for every patient. On average, each patient received two injections daily, one in the morning and one at noon. The injections are lumbar spinal nerve root analgesia (LSPA) of the affected nerve root in ‘freehand technique’^8^ Additional treatment consisted of 1 injection of the facette joints under x-ray and 1 epidural injections in ‘loss-of-resistance technique’ per stay. LSPA injections only contained 0.5% Scandicaine, whereas epidural injections and facette joint injection also contained triamcinolone 40 mg (Table 2)^3^,^9^,^10^,^11^. In cases of good effect the injection was repeated once.

Physiotherapy and sports therapy as part of inpatient MPM includes group exercises and aqua training; accompanying measures consist of electrotherapy for muscle relaxation and thermotherapy. In addition, patients are instructed in progressive muscle relaxation according to Jacobsen and take part in coordination training. The most effective exercises are isometric exercises for strengthening the back muscles, which is further aided by medical training therapy with workout equipment. The main goal is recovery of the load-bearing capacity and reduction in pain-avoidance behaviour. The success of MPM depends on accurate patient information and consultation, continuous motivation, a systematic increase of load and permanent feedback^12^.

The psychotherapeutic programme of multimodal pain management^13^ consists of an individual interview, but mainly of group therapy. Group therapy aims at motivating group members by introducing a new patient to the group discussion, thus raising the awareness of all group members. All patients have to keep a pain diary. The benefits of these therapy elements are well documented^14^,^15^,^16^. The aim is to mediate disease-specific information, promote health and vitality and decrease psychophysical activation by stress factors, especially pain (Table 3)^17^.

### Follow-up

Data were recorded in standardised form at baseline and at the 1-year follow-up. The data obtained before and after treatment were compared to assess treatment success. The data provided information on the length and extent of the treatment success 1 year (±2 weeks) after hospitalisation. Physical and psychological factors were evaluated by means of the Oswestry Disability Index (ODI), the Short Form-36 (SF-36), the numerical rating scale of pain (NRS) and the Hospital Anxiety and Depression Scale (HADS-D). A detailed medical history and clinical examination of the patient completed the data for the assessment.

### Statistical analysis

Data were compared with the t-test for paired variables and the non-parametric Wilcoxon test. The level of significance was set at p < 0.05. Statistical analysis was done with the programme SPSS 19.0 by IBM®.

## Results

In all patients, the diagnosis of lumbar radiculopathy was made by means of patient history and clinical symptoms prior to treatment. For 59 patients, an additional clinically relevant finding was detected in the MRI (VA herniation or protrusion). Only 1 patient had no radiological finding corresponding to the symptoms but still met the indication for MPM. As expected, L4/L5 was the most commonly affected site in our study population (Table 4).

### Pain

**Figure 2:**
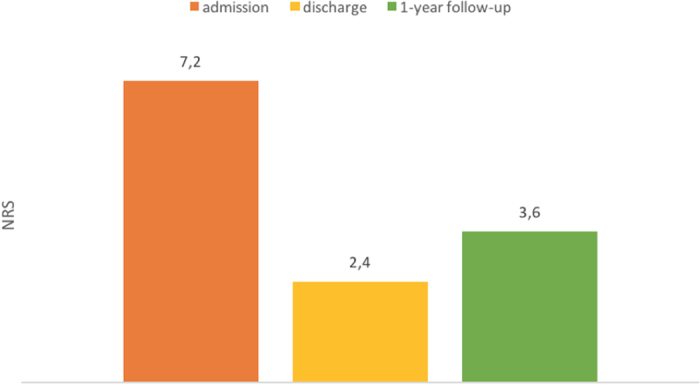
Mean values of NRS at admission, after discharge and at 1-year follow-up.

The mean NRS value was 7.2 before treatment, 2.4 at discharge and 3.6 at follow-up, as shown in Fig. 2. 100% of the patients showed a short-term reduction and 87% a mid-term reduction in NRS values and therefore in pain (Fig. 2).

**Figure 3:**
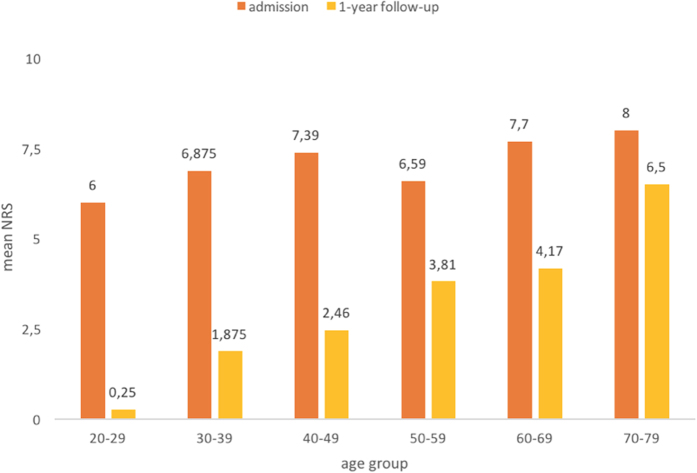
Mean NRS values according to age group.

Dividing patients into age groups on average shows lower NRS values for younger patients at follow-up (Fig. 3).

### Flexibility

Finger-floor distance had significantly improved from a mean of 20.92 cm (SD: 18.921) before treatment to 10.1 cm (SD: 12 097) at the 1-year follow-up (p < 0.05). Another spinal mobility measure is testing the motion directions to detect any mobility restrictions. At the initial examination, 37 patients showed mobility restriction in at least one direction. This figure was significantly reduced to 18 patients at follow-up (p < 0.05).

### Senso-Motoric Development

Motor deficits in the sense of serious paresis such as foot drop were not included here because such deficits represent an indication for surgical treatment, which would have resulted in the withdrawal from the study. At the initial examination, 10 patients had motor deficits in terms of reduced strength (3/5 or 4/5) but none at follow-up. This difference is significant (p < 0.05). Sensory deficits such as hyposensitivity or paraesthesia were reduced from 25 to 17. 14 patients still experienced hyposensibility at different degrees, and 3 patients had paraesthesia. The number of complaint symptoms could also be reduced, and, according to the hypothesis, the difference was statistically significant in a one-sided test (p < 0.05). Thus, MPM reduced motor and sensory deficits as well as pain and accompanying symptoms.

### Questionnaires

The Oswestry Disability Index (ODI) is used to measure everyday impairment. The mean value in our patients at the initial examination was 38.95% and 22.83% at follow-up, hence the reduction was significant (p < 0.05). In addition, the frequency of category ‘0% to 10%’ could be increased from 0 at baseline to 16 at follow-up. The Hospital Anxiety and Depression Scale (HADS-D) was additionally used to detect possible psychological impairment and to gain a comprehensive picture of a patient’s situation. No significant difference was found, only a slight decrease in the positive results. Nevertheless, 4 positive results were found at follow-up. Patients with a positive anxiety value often also showed a positive depression value. Thus, only a few patients had slightly elevated results in the HADS-D (n = 8 before treatment, n = 8 after treatment).

The Short Form 36 (SF-36) was used in addition to the ODI and the HADS-D. The SF-36 and the ODI not only record physical impairment but – similar to the HADS-D − also issues of mental health. Thus, only the following 3 parameters were used when evaluating the SF-36: ‘subjectively experienced current state of health’, ‘current impairment’, which was categorized, and ‘health status compared to last year’. The information given on the perceived health status showed its development from the initial examination to follow-up. The median of this topic had significantly changed from ‘less well’ at baseline to ‘good’ at follow-up (p < 0.05). As already indicated by the ODI, even everyday impairment could be significantly reduced (p < 0.05). The majority of patients (n = 37; 63%) rated their own health as improved, and only 8 patients considered their own health deteriorated over the previous year. However, some of these patients had developed other diseases and traumas such as a femoral neck fracture in addition to their back problems.

### Patient Satisfaction

At follow-up, patients were asked how satisfied they were with the success of their treatment and the treatment plan. 35% of patients (n = 21) were very satisfied, 52% (n = 31) were satisfied and 13% (n = 8) had an impartial attitude regarding the success and concept of the treatment. If required, most patients would repeat the therapy and recommend the treatment. Patients who did not reach the desired medium-term treatment outcome did still not view the treatment in a negative manner because the initial pain relief was perceived as very positive. In addition, patients had perceived the treatment and care within the MPM concept as very good and pleasant.

## Discussion

Discogenic disorders mostly occur at certain levels of the spine. Segments L4/5 and L5/S1 and their respective nerve roots, for instance, are mostly stressed mechanically and therefore most commonly affected^18^. This fact could also be seen in the patients of this study. The nerve roots L4 (10%), L5 (56.7%) and S1 (28.3%) were affected corresponding to the pathological findings at the described levels. A similar distribution was also shown in a study by Carette *et al*.^19^. Here, the L4 dermatome was affected in 13% and the L5 dermatome in 46.2% of the cases. Many studies have shown the high potential for spontaneous remission^20^,^21^.

As conservative treatment options usually only single methods are compared. There are either only injections^22^ or different types of physiotherapy^23^ compared to surgery or waiting for remission because of natural history. All these single methods need more time for a longer lasting success. Especially injections alone are known for only short term success^24^. Another problem is the low frequency of treatment for outpatient patients. Therefore, it takes longer to have the first pain relief and there is a high danger to have pain chronification.

There we see the big advantage of MPM. Patients get a lot of different conservative treatment methods in a very short time period. This leads to a quick pain relief and in combination with the psychological lessons avoids chronification.

In contrast to former opinions, today pain management and physical activation are the centre of conservative treatment. The aim of the accompanying methods is to preserve or restore the full capacity of the spine-stabilizing muscles, to train coordination skills and to learn spine friendly behaviour. Risk factors that can lead to chronic pain must be eliminated. All methods serve to interrupt the vicious circle of “pain - stress - malposition- pain”.

The injections help to reduce pain as fast as possible^25^,^26^. Then patients can take part in activating treatments like back exercises or aqua training more intensively due to reduced pain. Beside direct physical effects the accompanying therapies also help to improve the affective-emotional level as well as the motor-psychomotor level by learning physically regulating movements. The focus is on activating methods, not on passive methods.

So chronification can be avoided and when being back in daily life, the patient has new possibilities to avoid falling back into the vicious circle of pain and has the positive feeling that there are always treatment options to reduce pain. For us this is the reason for the very good med-term effects of the MPM.

The variety of pain scores used in the different treatment concepts makes a comparison with other studies difficult. Even comparisons within one and the same study are often not possible because answers are always subjective assessments, although items are objectified by pain scales. Thus, only comparisons are useful that include the scores before and after treatment and that are carried out for each individual patient. In the present study, a numerical pain scale was used, and its classification ranged from 1 to 10. The initial examination yielded values between 4 and 10 and an average value of 7.2. At discharge, the values ranged between 0 and 6, and the average value was significantly reduced to 2.4 (p < 0.05). All patients experienced at least temporary pain reduction. At the 1-year follow-up, values ranged between 0 to 10, and the average value was 3.6, which showed significant pain reduction (p < 0.05). Particularly younger patients had lower NRS values in the follow-up, thus benefited most from MPM. This finding is probably due to the fact that younger patients are not yet affected by degenerative changes, which are likely to contribute to pain symptoms.

Ultimately, the pain symptoms of the patients in the present study could be reduced on average by 50%. However, because a ordinal scale was used in this study, it cannot be assumed that average pain intensity was halved. Spinal mobility was determined by means of finger-floor distance and movement restrictions according to active and passive mobility. However, not only restricted mobility of the lumbar spine may impede finger-floor distance but also shortened hamstrings and limited hip function. Nevertheless, a radiological study showed a valid connection between finger-floor distance and lumbar spinal mobility^27^. Finger-floor distance had significantly decreased to 10.10 cm on average at follow-up in contrast to 20.92 cm at the initial examination (p < 0.05).

Passive and active mobility provides more information on spinal mobility and possible spinal injury. Examinations test lateral flexion and rotation as well as restrictions in inclination and reclination angles which are often impeded in the presence of lumbar pain. At the initial examination, 62% of the patients in the current study had restricted mobility in at least one direction. Until follow-up, this figure could be reduced to 30% (p < 0.05). Thus, a significantly lower value (p < 0.05) was achieved in both mobility restriction and finger-floor distance.

The case-control study by Thomas *et al*.^28^ showed a significant correlation between back pain and reduced spinal mobility. The group of patients with back pain had significantly worse spinal mobility than the control group. The collected data of the present study could also show this correlation. The spinal mobility of patients could be considerably improved by MPM.

In addition to pain, patients are particularly affected by motor and sensory deficits. For this reason, attention should also be focussed on these complaints, although they are usually not a patient’s prime concern. Pain and its treatment are usually the most important issues for affected patients. At baseline, 10 patients of the present study had a motor deficit such as minor reduction in strength that did not indicate surgery. At follow-up, none of the patients had any motor skill problems. Thus, motor deficits could be reduced by 100%, which it is a significant result (p < 0.05). In addition, 25 patients had complained about sensitive deficits at baseline. At follow-up, this figure was reduced to 17. 14 patients still experienced hyposensibility at different degrees, and 3 patients had paraesthesia. However, these deficits were only intermittent. Of further significance is also the successful treatment of symptoms in 8 patients (p < 0.05). MPM resulted in significant pain relief (p < 0.05) as well as in decreased accompanying motor and sensory deficits. A similar development was also found by Madl *et al*.^1^. The Oswestry Disability Index (ODI) serves to objectify everyday impairment, thus values between 0% and 100% are possible. The higher the value, the higher is the impairment in everyday life. In the present study, ODI was collected at baseline and at follow-up. At the time of discharge, no ODI data were collected. The mean value at the initial examination was 38.95% and 22.83% at follow-up, hence the reduction was significant (p < 0.05). In addition, the frequency of category ‘0% to 10%’ could be increased from 0 at baseline to 16 at follow-up. Besides pain reduction, the limitations in daily life could be also be reduced, mainly because of the strong correlation between pain and impairment in daily life. Lumbar radiculopathy impedes everyday actions such as getting dressed. A decrease in pain therefore reduces everyday impairment. The concept of MPM takes advantage of this fact by starting physiotherapeutic exercises at this point.

The SF-36 questionnaire was used in addition to the ODI and the HADS-D because it measures both physical and psychological impairment. Therefore, only the following three parameters were used: ‘subjectively experienced current state of health’, ‘current impairment’, which was categorized, and ‘health compared to last year’. The patients’ current health status was documented in categories. The median had changed from ‘less good’ to ‘good’ from the first examination to the follow-up. Categorized impairment showed of course a similar pattern of that already provided by the ODI. Again, the number of patients with severe to very severe impairment had been reduced from 10 to 4. At the same time, the number of patients with little or no impairment had significantly increased from 3 to 26. Patients were also asked about the development regarding their general state of health. 63% of patients felt better 1 year after the therapy than at the beginning. Only 14% of patients reported deterioration in health. However, since this question included the development of general health and not just of back problems, the informative value of this parameter regarding therapeutic success is limited. For example, some patients had meanwhile been diagnosed with an internal disease or with a femoral neck fracture.

Subjective patient satisfaction reflects very well how obtained results correspond with patient demands and expectations. So what do patients demand and expect from this treatment method? Demands and expectations may range from pain relief to slight improvement to maintaining the current health status. In the present study, 35% of patients were very pleased, 52% were pleased and 13% had an impartial attitude regarding the success and concept of the treatment. These figures may seem at first a bit surprising, as some patients still experience pain, partly also of higher intensity than before the treatment. Nevertheless, none of the patients commented negatively on the treatment which may be due to several reasons. As already mentioned, patient satisfaction crucially depends on patient expectation. Even small improvements of often short duration may already be perceived as positive, thus influencing patient satisfaction. Furthermore, the care provided, the detachment from the challenges of daily life and the related psychological relief may be perceived as positive by the patients^5^. These factors were stated by many patients in the current study. Despite attempts to exclude any iatrogenic influence, patients may have been somewhat influenced when answering this question because of the principle of social desirability.

## Conclusion

The lack of a control group is a limiting factor of the study. But evaluating the overall concept of MPM and not just the sub-item of injections makes the implementation of a control group difficult. Moreover, study populations may show a trend towards chronic back problems because the admission requirement of inpatient multimodal therapy is the failure of outpatient unimodal therapy. Despite these limitations, this study yielded satisfactory results. Treating radicular pain by inpatient MPM based on injections still shows an average pain reduction by 3.6 points 1 year after therapy. The positive picture is completed by the subjective impression of the patients: 87% of the patients were satisfied or very satisfied with the treatment. 87% of the patients achieved medium-term pain relief. Finally, 12.98% of all treated patients had to be operated on during the observation period because of radicular and pseudoradicular complaint pathologies. In addition, no serious complications were observed.

In summary, MPM based on injections is an effective and low-risk treatment option for lumbar radiculopathy. In the absence of an absolute indication for surgery or an absolute contraindication for MPM, patients with lumbar radiculopathy should be first treated with this minimally invasive intervention.

### Ethical approval and informed consent

The study was approved by the Ethics Commission of the University of Regensburg and carried out in accordance with the approved guidelines. Registration in DRKS number DRKS00010257.

## Additional Information

**How to cite this article**: Benditz, A. *et al*. Prospective medium-term results of multimodal pain management in patients with lumbar radiculopathy. *Sci. Rep.* **6**, 28187; doi: 10.1038/srep28187 (2016).

## Figures and Tables

**Table 1:** Antropometric data of the patient group (mean and range).

	**Female** (**n** = **26**)	**Male** (**n** = **34**)	**Together**
Age (years)	56,35 (33–77)	51,15 (29–79)	53,40 (29–79)
Height (centimetres)	163,56 (156–173)	177,42 (156–195)	172,53 (156–195)
Weight (kilogram)	76,44 (55–105)	90,80 (69–125)	85,74 (55–125)
BMI	28,55 (21,48–37,80)	28,66 (23,14–40,49)	28,62 (21,48–40,49)

**Table 2:** Overview of injections.

	**Minimum**	**Maximum**	**Average**	**Standard deviation**
LSPA	10	24	17,75	2,634
Epidural injections	0	5	1,7	0,809
Facette injections	0	3	1,13	0,769
Iliosacral joint injection	0	3	0,22	0,585

**Table 3:** Example of a 10-day schedule of physiotherapy and sports therapy as part of inpatient MPM.

**exercise**	**number**
Group exercises	4
Aqua training	7
back exercises	5
Instructions for progressive muscle relaxation	3
Coordination training group	4

**Table 4:** Frequency distribution of the affected nerve root at the start of treatment.

**Nerve root**	**L2**	**L3**	**L4**	**L5**	**S1**
absolute frequency	2	1	6	34	17
Frequency in percent	3,3%	1,7%	10%	56,7%	28,3%
